# The relationship between mitral annular systolic velocity and ejection fraction in patients with preserved global systolic function of the left ventricle

**DOI:** 10.1186/1471-2261-13-92

**Published:** 2013-10-26

**Authors:** Ivaylo Rilkov Daskalov, Ivona Kirilova Daskalova, Lilia Davidkova Demirevska, Borislav Georgiev Atzev

**Affiliations:** 1 Department of Cardiology and Intensive Care, Cardiology Clinic, Military Medical Academy, 3 Georgi Sofiiski Blvd, Sofia 1606, Bulgaria; 2 Department of Endocrinology and Metabolic Disorders, Endocrinology, Military Medical Academy, 3 Georgi Sofiiski Blvd, Sofia 1606, Bulgaria; 3 Department of Cardiology, University Hospital “St.Ekaterina”, 52A Pencho Slaveikov Blvd, Sofia 1000, Bulgaria

**Keywords:** Mitral annulus velocity, Ejection fraction, EF, Systolic function, Sm, TDI

## Abstract

**Background:**

The aim of the study was to investigate the relationship between the ejection fraction (EF) and the mitral annular systolic velocity (Sm) in patients with preserved left ventricular systolic function (EF>55%). The study task was to evaluate whether the assessment of Sm(avg) can be used as an alternative to the Simpson’s method in assessment of the EF. The expected benefit was that Sm could be used to predict EF, when EF is difficult to assess due to poor image quality (IQ).

**Method:**

Sm was obtained by spectral pulse wave Tissue Doppler Imaging (pwTDI) from the lateral and septal sites of the mitral annulus (MA) and an averaged value was calculated - Sm(avg). EF was assessed using Simpson’s rule. Participants were divided into controls (n=70), hypertensive (HTN, n=56), HTN with diastolic dysfunction (HTN/DD, n=65), HTN with diabetes mellitus (HTN/DM, n=52) and HTN with DD and DM (HTN/DD/DM, n=65).

**Results:**

Sm(avg) showed strong correlation with EF (r=0.978; p<0.0001). There were no significant differences between the correlation coefficients between the subgroups and the controls. The mathematical model that the study recommended to assess the EF is: *EF=45.0 + 2 × Sm(avg).*

**Conclusion:**

The assessment of Sm(avg) could be used as an alternative to EF. This approach may be useful especially when the IQ is poor. The method maintains high accuracy and reproducibility in prediction of the EF.

## Background

Left ventricular (LV) longitudinal shortening during ejection reflects mitral annulus (MA) descent and has been used as an index of the global systolic function [1-5]. Previous studies using routine M mode, two-dimensional echocardiography and colour-coded M mode TDI measures of MA systolic excursions have shown close correlation with EF which is an accepted standard reference of LV systolic function [6-8]. The measurement of MA systolic velocities (Sm) is advantageous compared to the other quantitative echocardiographic techniques, because it is not dependent on the endocardial definition [9]. This parameter, measured by pwTDI, correlates more strongly with plasma BNP levels than those measured by M-mode. It provides a simple, sensitive, accurate and reproducible tool for early diagnosis of LV dysfunction [10].

Even though Sm of the MA correlates closely with the EF, they are not directly connected [10]. The relationship is probably nonlinear in patients with reduced EF in contrast to the subjects with preserved EF where the relationship is linear. Many factors influence the EF/Sm(avg) ratio, such as gender, age, presence and grade of hypertension (HTN), diastolic dysfunction (DD), coronary heart disease (CHD), diabetes mellitus (DM) etc [11,12].

### Objective

The aim of this study was to investigate whether the assessment of Sm(avg) can be used as an alternative to the Simpson’s method in assessment of the EF. The study task was to find an equation from which EF could be predicted. The benefit of this approach is that Sm is not dependent on IQ and therefore could apply to subjects with poor IQ.

## Method

The study protocol was approved by the local Ethics Committee of the Military Medical Academy Sofia Bulgaria. All participants agreed and signed the informed consent form of the study. Subjects were randomly selected between January 2009 and August 2011 from outpatient clinic and hospitalized patients at the Department of Cardiology and Intensive Care.

Assessment of participants was performed by experienced cardiologists using a standard protocol, including questions on medical history, family history, cardiovascular risk factors, alcohol intake, physical activity and drug history. A physical examination which included blood pressure, anthropometric measurements, and an electrocardiogram was performed. Standard laboratory blood tests were performed to identify subjects with DM (fasting blood glucose, HbA1C), dyslipidemia (cholesterol, LDL, HDL, TG), anemia (Hb), significant liver (AST, ALT, GGT, bilirubin) and kidney disease (creatinine, urea). Subsequently, eligible subjects were invited to undergo echocardiography. Echocardiography was performed in a left lateral decubitus position, with a digital commercial harmonic imaging ultrasound system, АLOKA PROSOUND SSD5500 SV, equipped with a 2.5 MHz phased-array transducer. The images were acquired during a breath hold. Left ventricular dimensions were obtained in the parasternal short axis view, and LV mass was calculated using the Devereaux formula and indexed to height to give the LV mass index (LVMI). Left ventricular hypertrophy was investigated by LVMI and the thickness of the walls. The EF was assessed using the biplane Simpson’s method [13]. Left atrial volume was calculated from 3 measurements of left atrial dimensions using the formula for an ellipse and indexed to body surface area to obtain the left atrial volume index (LAVI). Diastolic function of the LV was assessed using the following indices: ratio E/A, Valsalva maneuver ΔE/A, ratio E/e’, LAVI, pulmonary artery systolic pressure (PAS), IVRT/T _E-e’_ and Ar-A [14].

**Figure 1:**
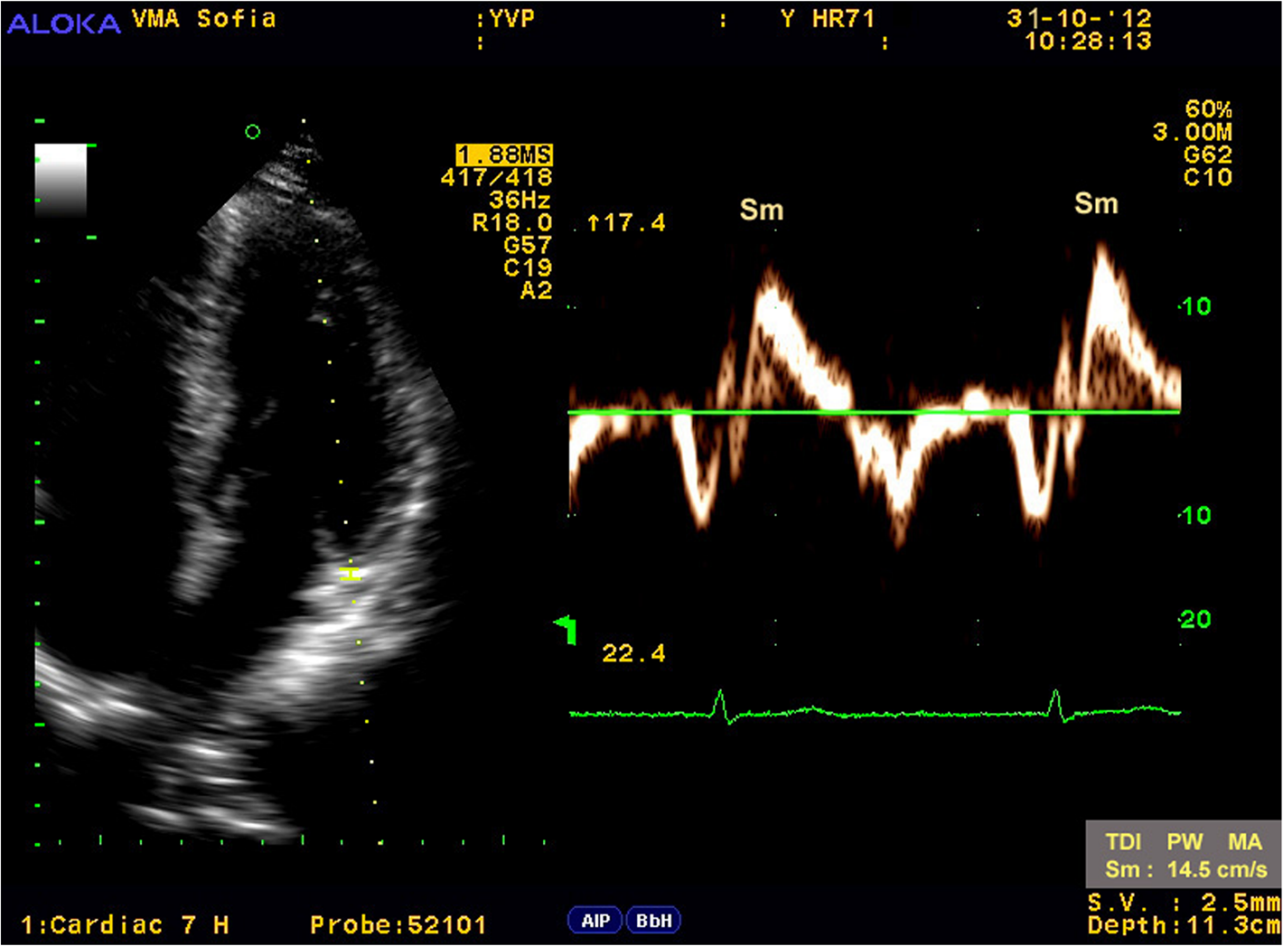
The picture presents how to obtain Sm from the lateral site of the MA using spectral pwTDI.

**Figure 2:**
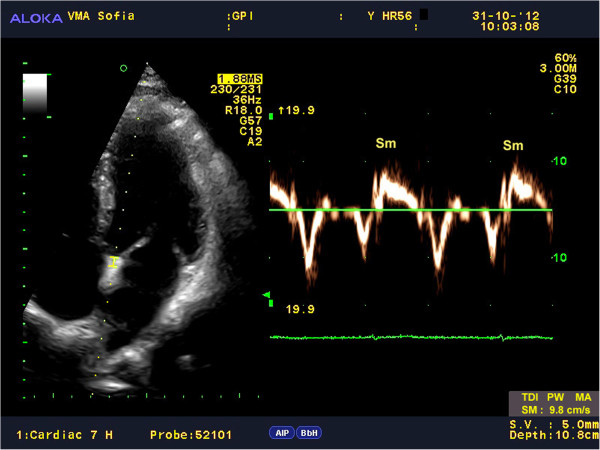
The picture presents how to obtain Sm from the septal site of the MA using spectral pwTDI.

Myocardial velocities were measured on-line using spectral pwTDI [15,16]. The sample volume was guided by color-coded images, which were acquired using low velocity, high-intensity myocardial signals at a high frame rate (>150 MHz). The imaging angle was adjusted to ensure as near to a parallel alignment of the beam as possible with the myocardial segment of interest. Longitudinal contraction of the LV was investigated by average peak systolic velocity of the mitral annulus – Sm(avg) using two positions, septal and lateral, from the apical 4-chamber view (Figure 1, Figure 2).

In accordance with the study protocol, 3 consecutive cardiac cycles were analyzed, and the mean value was calculated. After each evaluation, the results were processed with the АLOKA D4D software and digitally stored (post-processing) to evaluate the reliability of the results. Two experienced echocardiographers evaluated the results of 10 randomly selected participants from every group, independently from one another, for the analysis of intra- and inter-observer variability.

### Statistical analysis

The statistical analysis was performed using SPSS (Statistical Package for Social Science) version 15.0 for Windows. The significance level for this study was set at *p* < 0.05. Categorical data were shown by frequencies and percentages. ANOVA was used to analyze the demographics and clinical characteristics of the study population. We performed the analysis in subgroups using an unpaired t-test. To determine the strength of the correlation between the variables, we used the Pearson’s **r** correlation coefficient. Continuous variables and their association with age, gender, HTN, DD and DM were assessed using multiple regression analysis. Reference ranges are presented as values denoting the 95% confidence interval for correlations. The mathematical model was created by linear regression analysis. Inter- and intra-observer variability of Sm(avg) and EF were assessed using intraclass correlation coefficients (ICC).

## Results

Participants were patients with hypertension (HTN, n=56), HTN and diastolic dysfunction (HTN/DD, n=65), HTN and diabetes mellitus (HTN/DM, n=52) and patients with HTN, DD and DM (HTN/DD/DM, n=65). All these patients were compared with a control group consisting of age-matched healthy subjects (controls, n=70). All the patients were in sinus rhythm and had a preserved EF (>55%). There were no patients with signs and symptoms of coronary heart disease (CHD), or more than a mild valvular heart disease (Table 1).

**Table 1:** Demographics and clinical characteristics of the study population

***Participants (n=308)***	***Controls (n=70)***	***HTN pts. (n=56)***	***HTN/DD pts. (n=65)***	***HTN/DM pts. (n=52)***	***HTN/DD/DM pts. (n=65)***	***p-value***
Gender/male (n, %)	40 (57.1%)	30 (53.5%)	30 (46.2%)	28 (53.8%)	32 (49.2%)	0.899
Mid age (years)	*45*±10	53±10	56±10	55±10	60±10	0.462
Height (cm)	170±14	168±16	168±14	169±13	165±14	0.241
Weight (kg)	66±7	67±9	65±9	69±9	68±9	0.311
BMI (kg/m^2^)	22.9±2	23.3±2	23.1±2	24.5±2	24.1±2	0.233
Heart rate (bpm)	70±10	72±10	68±10	64±10	65±10	0.08
Systolic BP (mmHg)	125±5	128±5	130±5	118±5	120±5	<0.0001
Diastolic BP (mmHg)	70±8	78±8	80±8	77±4	76±3	<0.0001
EF (Simpson’s method %)	64.4±2%	63.7±4%	63.9±4%	64.9±4%	62.9±4%	0.161
Fasting blood glucose	4.8±1.0	5.0±1.0	5.0±0.7	6.0±1.5	7±1.5	<0.0001
HbA1c	4.0±1.0	4.5±1.0	4.7±1.0	5.0±1.0	5.3±1.0	<0.0001
DM type II	0	0	0	29 (55.5%)	30 (46.2%)	<0.0001
HTN mild	0	22 (39.3%)	23 (35.3%)	20 (39.4%)	25 (38.5%)	<0.0001
HTN moderate	0	18 (32.1%)	25 (38.5%)	23 (44.2%)	27 (41.5%)	<0.0001
HTN severe	0	16 (28.6%)	17 (26.2%)	9 (17.3%)	13 (20%)	<0.0001
DD impared relaxation	0	0	35 (53.8%)	0	34 (52.3%)	<0.0001
DD peudonarmalisation	0	0	20 (30.7%)	0	20 (30.7%)	<0.0001
DD restriction	0	0	10 (15.4%)	0	11 (16.9%)	<0.0001

The aim of the study was to establish a quantitative method for evaluation of EF using Sm(avg). This approach presents an opportunity to predict EF by using an equation. In this equation the main independent variable is Sm (avg), which is obtained by spectral pwTDI.

The quantification of EF by equation has an advantage that Sm is minimally dependent on IQ, as well as a disadvantage that the mathematical model requires memorization of the two constants.

**Figure 3:**
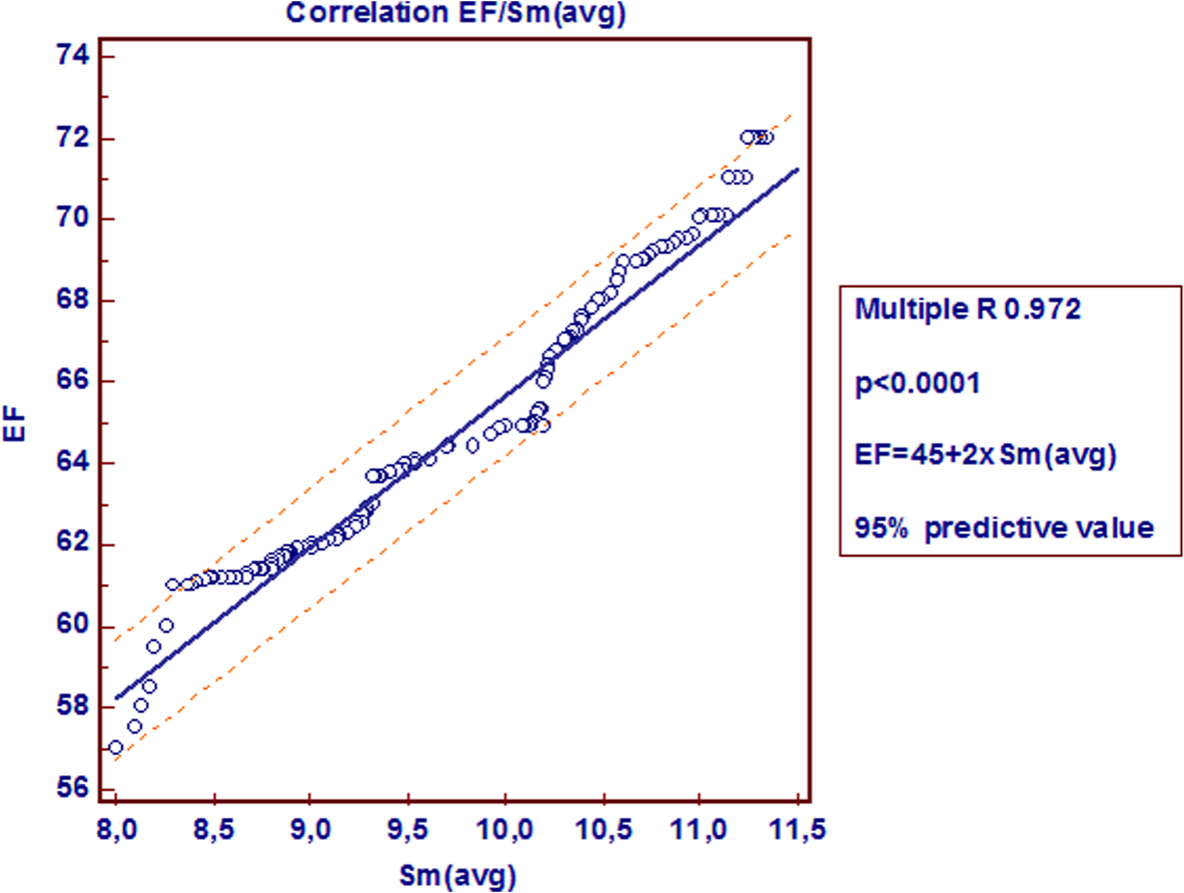
The scatter diagram presents a regression line, as a result of the linear correlation between EF and Sm(avg).

This study demonstrated a linear correlation between EF and Sm(avg) for investigated subjects with preserved global systolic function (Figure 3).

The multiple regression analysis demonstrated strong correlation between investigated variables. There was no significant difference between the subgroups regarding the strength of the correlation. Therefore it was possible to recommend a general mathematical model for the entire study population (Table 2).

**Table 2:** Multiple regression analysis, correlation coefficients, significance, 95% confidence interval standard deviation and mathematical model

***Number of patients (n=308)***	***Correlation coefficient (R)***	***95% confidence interval (CI)***	***Asymptotic significance (p)***
Multiple correlation EF/Sm(avg)	0.978	0.975–0.980	< 0.0001
Controls (n=70)	0.936	0.932–0.938	< 0.0001
HTN (n=56)	0.882	0.880–0.886	< 0.0001
DD (n=65)	0.906	0.902–0.910	< 0.0001
DM (n=52)	0.948	0.942–0.950	< 0.0001
HTN/DD/DM (n=65)	0.963	0.960–0.968	< 0.0001
	*Meaning*	*Standard Deviation (SD)*	
Constant	45.0	±1.882	p < 0.0001
Coefficient	2.0	±0.191	p < 0.0001
Equation		***EF = 45.0 + 2 ***_***X ***_ ***Sm(avg)***	

Many factors influence the relationship of EF/Sm(avg) and could change the defined equation. These are: gender, age, HTN, DD, DM, CHD, anemia, chronicle renal diseases etc. In this study only the influences of gender and age, HTN, DD and DM were investigated. The data demonstrated that gender and age influence Sm(avg) in a way that higher velocities are observed in men while they decrease linearly with age. However, changes of the EF/Sm(avg) correlation were not significant; therefore there was no need to correct the defined mathematical model (Tables 3 and 4).

**Table 3:** The influence of gender on the EF/Sm(avg) correlation and Sm(avg) velocity

**Gender (n=308)**	**Men (n=160)**	**Women (n=148)**	
	**Pearson’s correlation**	**Pearson’s correlation**	**Significance**
EF/Sm(avg)	r=0.906 (p=0.002)	r=0.881 (p=0.004)	*p=0.279*
Sm(avg)	10.3±0.9 (cm/s)	9.2±0.12 (cm/s)	*p<0.0001*
EF (%, mean, ± SD)	66 ±2	63±3	*p<0.0001*

**Table 4:** The influence of age on the EF/Sm(avg) correlation and Sm(avg) velocity

**Age/years (n=308)**	***20–40 (n=75)***	***41–60 (n=123)***	***61–80 (n=110)***	***Significance***
	**Pearson’s correlation**	**Pearson’s correlation**	**Pearson’s correlation**	
*EF/Sm(avg)*	r=0.978 (p=0.002)	r=0.934 (p=0.044)	r=0.889 (p=0.02)	*P=0.625*
*Sm(avg)*	11.2±0.1 (cm/s)	9.6±0.1 (cm/s)	8.6±0.1 (cm/s)	*P<0.0001*
*EF* (%, mean ±SD)	67±2	64±2	62±2	*P<0.0001*

The data demonstrated that HTN, DD and DM influence Sm(avg). In patients with HTN without DD there was a tendency to weaken the EF/Sm(avg) correlation. In subgroup with HTN/DD, we observed a reverse tendency. In patients with HTN/DM correlation was stronger. These tendencies did not differ significantly between subgroups. Therefore, it was not necessary to correct the general equation. Regarding Sm(avg), there was a tendency to decrease the velocity in each group, depending on the grade of HTN, DD and/or DM. Combination of these conditions increased the severity of Sm(avg) deceleration.

According to our data, we can state that there was a significant decrease of Sm(avg) velocity (p<0.04), which differentiates controls from other subgroups. Subjects with HTN define a cut-offs (<10.2cm/s) as a result of Sm deceleration, which depends on the grade of HTN and co-morbidity. This deceleration could be mild (HTN), moderate (HTN/DD, HTN/DM) or severe (HTN/DD/DM) (Table 5).

**Table 5:** The influence of HTN, DD and DM on EF/Sm(avg) correlation and Sm(avg)

**Participants (n=308)**	***Controls (n=70)***	***HTN (n=56)***	***HTN/ DD (n=65)***	***HTN/DM (n=52)***	***HTN/DM/DD (n=65)***	***p*-value**
*Pearson’s* ***(r)***	0.936 (p=0.030)	0.882 (p=0.002)	0.906 (p=0.045)	0.948 (p=0.004)	0.963 (p=0.022)	*0.543*
*Sm(avg)* (cm/s)	10.4–10.2	10.2–9.9	9.8–8.8	10.2–8.8	8.8–8.3	*0.04*
*EF* (%, mean ±SD)	66±2	65±2	64-62	65-62	62-61	*<0.0001*

To assess the reliability of the measurements, we used intraclass correlation coefficients (ICC). Two experienced echocardiographers evaluated the results of 10 randomly selected subjects from every subgroup, independently from one another, for the analysis of intra- and inter-observer variability.

Analyzing the results, we could state that Sm(avg) and EF demonstrated better than good reliability for one as well as for two independent investigators. It is important to note that there were small fluctuations in the strength of agreement between subgroups and within groups. These changes reflected realistic variability of the measurements, depending on co-morbidity, gender and age, and, mostly, on the quality of echo images. In healthy subjects, both methods were equivalent in assessing the global systolic performance. In all others subgroups there was a prevalence of TDI obtained Sm(avg). This was strong evidence for the benefits of the spectral pwTDI whenever the assessment of EF (by Simpson’s rule) is problematic due to poor IQ (Table 6).

**Table 6:** Assessment of the reliability of the measurements of EF and Sm(avg) by ICC

**Participants (n=308)**	**ICC (1 observer)**	**95% CI**	**Strength of agreement**	**ICC (2 observers)**	**95% CI**	**Strength of agreement**
**Controls** (n=10)	*Single measures*			*Average measures*		
EF (%)	0.9337	0.9020 ÷ 0.9553	Very good	0.9653	0.9485 ÷ 0.9772	Very good
Sm _(avg)_ (cm/s)	0.8634	0.8010 ÷ 0.9010	Very good	0.9216	0.8901 ÷ 0.9450	Very good
**HTN** (n=10)						
EF (%)	0.6337	0.6020 ÷ 0.7553	Good	0.7653	0.6485 ÷ 0.7772	Good
Sm _(avg)_ (cm/s)	0.8337	0.8015 ÷0.8080	Very Good	0.9059	0.8095 ÷ 0.9210	Very Good
**HTN/DD** (n=10)						
EF (%)	0.6841	0.6224 ÷ 0.7509	Good	0.7790	0.6183 ÷ 0.7894	Good
Sm _(avg)_ (cm/s)	0.7838	0.7131 ÷ 0.8121	Very Good	0.8254	0.8111 ÷ 0.9211	Very Good
**HTN/DM** (n=10)						
EF (%)	0.6549	0.6338 ÷ 0.7009	Good	0.6919	0.6759 ÷ 0.7479	Good
Sm _(avg)_ (cm/s)	0.7625	0.7346 ÷ 0.8434	Very Good	0.7784	0.8115 ÷ 0.9300	Very good
**HTN/DD/DM** (n=10)						
EF (%)	0.6841	0.6324 ÷ 0.7509	Good	0.6790	0.6183 ÷ 0.7194	Good
Sm _(avg)_ (cm/s)	0.7945	0.7234 ÷ 0.8222	Very Good	0.8854	0.8005 ÷ 0.9401	Very good

The ICC is a measure of the reliability of measurements or ratings. The table reports two coefficients with their respective 95% CI. Single measures: this is an index for the reliability of the ratings for one, typical, single rater and average measures which is an index for the reliability of different raters averaged together. (<0.20 Poor; 0.21-0.40 Fair; 0.41-0.60 Moderate; 0.61-0.80 Good; 0.81-1.00 Very good).

## Discussion

This study evaluated the prediction of EF by Sm only in patients with preserved EF. Spectral pwTDI could be used as an alternative examination when EF is difficult to assess or the results are controversial. The study demonstrated a simple approach to predicting EF using Sm(avg) from spectral pwTDI.

This approach (quantification) is based on a linear equation where estimated Sm(avg) could be used to calculate EF. This is a simple, cheaper and faster method compared to cardiac MRI, CT or contrast echocardiography. The quantitative approach presents an opportunity to predict EF using an equation. This equation uses Sm(avg), as well as a constant and a coefficient. The method is clinically useful due to the minimal dependency of Sm on the IQ. In contrast, EF demonstrates strong dependency on the visibility of the endocardial contour when using Simpson’s method.

Regrettably, clinical practice shows that the use of mathematical models has a few evident disadvantages. First of all, equations are not very well accepted due to the need of memorizing many constants. These constants always generate standard error of estimation, which cannot be avoided and should be kept in mind. Furthermore, these equations are rarely included in the software of the echo machines.

For quantitative analysis of the EF, it could be applied to the general mathematical model: *EF = 45.0 + 2*_*X*_*Sm(avg).*

The study confirmed dependency of the longitudinal systolic contraction on gender and age. Most researchers supported the hypothesis that Sm decreases with age and in women [17-20]. Our study supported this conclusion.

Several studies have demonstrated that patients with HTN, CHD, DD and DM have lower systolic and early diastolic velocities in the MA compared to the controls [21-26]. Similar conclusions, especially for the systolic velocities, were presented in this study except CHD patients.

Finally, we have reached the general conclusion that regardless of the above-mentioned tendencies in patients with HTN, DD and DM, no correction in the explanatory equation is necessary. The reason is that no statistically significant differences between groups in EF/Sm(avg) correlation coefficients were observed.

One important limitations of this study was that the results were not valid for CHD patients and could not be used for them without further investigation. Another limitation was the lack of subgroups with atrial fibrillation, conduction abnormalities, pacemakers and prosthetic valves [27,28]. Therefore, we could not make any inferences about these specific conditions.

Another important limitation is that the correlation between EF and Sm in low ejection fraction was not examined. Therefore all results could be applied only to patients with preserved EF.

## Conclusion

The prediction of EF by Sm(avg) is a simple method, not time consuming, with high accuracy and reproducibility. The relationship between EF/Sm(avg) can be assessed by equation.

Finally, the mathematical model is valid only for a demonstrated subgroup of patients with preserved EF.

## Competing interests

The authors declare that they have no competing interests.

## Authors’ contributions

IRD and IKD planned the study. IRD and LDD investigated all patients, performed measurements and analyzed the data. IRD performed statistical analysis and wrote the manuscript. BGA, LDD and IKD made a critical review of the paper. All authors read and approve the final version of the manuscript.

## Pre-publication history

The pre-publication history for this paper can be accessed here:

http://www.biomedcentral.com/1471-2261/13/92/prepub
